# Modified Exfoliated Carbon Nanoplatelets as Sorbents for Ammonium from Natural Mineral Waters

**DOI:** 10.3390/molecules26123541

**Published:** 2021-06-10

**Authors:** Ion Ion, Daniela Bogdan, Monica Maria Mincu, Alina Catrinel Ion

**Affiliations:** 1 Department of Analytical Chemistry and Environmental Engineering, University Politehnica of Bucharest, 1-7 Polizu Str., 011061 Bucharest, Romania; ion.ion@upb.ro (I.I.); daniela.bogdan@gov.edu.ro (D.B.); 2 Horia Hulubei National Institute for R&D in Physics and Nuclear Engineering, 30 Reactorului Str., Magurele, 077125 Bucharest, Romania; monica.mincu@nipne.ro

**Keywords:** carbon nanomaterials, nitrogen species, natural waters

## Abstract

In this manuscript an improved sorbent based on modified exfoliated carbon nanoplatelets, applied in the removal of ammonium from aqueous samples, is presented. This sorbent showed better efficiency in comparison with the previous one obtained in our group for ammonium removal, the values of the maximum sorption capacity being improved from 10 to 12.04 mg/g. In terms of kinetics and sorption characteristic parameters, their values were also improved. Based on these results, a sorption mechanism was proposed, taking into account ion-exchange and chemisorption processes at the surface of the oxidized exfoliated carbon nanoplatelets. Future applications for simultaneous removal of other positive charged contaminants from natural waters might be possible.

## 1. Introduction

Among the indicators of purity in natural mineral waters, such as phenols, zinc and polyaromatic hydrocarbons (PAHs), a major one is ammonium, which is allowed to achieve a maximum concentration of 0.5 mg L^−1^. Endogenic sources of ammonium (NH_4_^+^) concern the hydrological, hydrogeological and hydro-chemical features of the aquifers [1]. Anthropogenic sources include agricultural wastewaters, nitrogen fertilizers and chemical plants [2]. In aqueous media, ammonium ions represent a source of other nitrogen compounds, such nitrites and nitrates, through oxidation processes, so its removal at a higher concentration than the permitted one is indicated. Especially nitrites can come only from ammonium ions present in the aqueous matrices, with maximum allowed concentrations of 0.1 mg L^−1^ in natural mineral waters. Nitrates can also come from the geological structure of the aquifer, in the absence of fertilizers and other nitrogen sources in exploited areas. 

**Figure 1:**
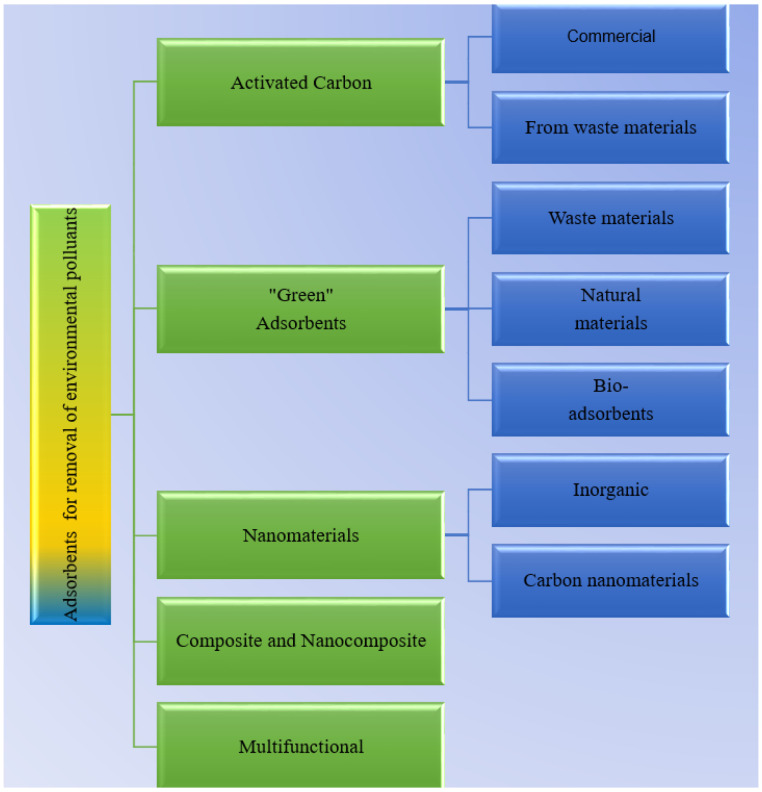
Carbon-based materials applied in environmental treatment of natural mineral waters.

Under the European Directive (EP 2009) [3], natural mineral waters are considered underground sources, few treatments that can affect their natural composition being admitted, except for filtration, oxygenation settling and treatment with ozone-rich air. This is why, in order to avoid any prohibited treatment, several sorbents [4] can be used in natural mineral water treatment, with activated carbon [5,6] being the most important one. As presented in Figure 1, in recent years, some nanomaterials have been replacing activated carbon [7,8,9], especially carbon-based nanomaterials [10,11,12]. These materials are proposed for the removal of some environmental contaminants based on their physical properties, such as high surface area and porosity. There are drawbacks due to the high costs of the activated carbon manufacturing process, but in the case of carbon nanomaterials, the low necessary amounts make them good candidates as sorbents in adsorption processes, especially when such nanomaterials are suitably functionalized. 

Oxidized carbon nanomaterials are obtained by using HNO_3_ [13], HNO_3_ + H_2_SO_4_ [14] and H_2_O_2_ + NH_4_OH [15]. The oxidized sites on the carbon surface can be identified and quantified by calorimetric analysis [16] and acid–base titration methods [17], discriminating the acidic functional groups that can control the chemisorption processes, such as alcohols, ketones and carboxylic groups.

Nitric acid can be used with the same concentration (65%), at the same temperature (140 °C), but at different refluxing times to oxidize the surface of carbon nanomaterials, certain differences being noticed among the concentrations of total acidic sites as a function of the reflux time [18]. To quantify the concentration of total acidic sites, Boehm’s titration [19], which selectively titrates the acidic sites with different acid dissociation constants, pK_a_, uses different bases with the right base pK_b_. As a result, NaOH solution is used as a titration reagent for carboxylic, ketone and phenolic groups, and NaHCO_3_ is used as a titration reagent only for carboxylic groups.

**Figure 2:**
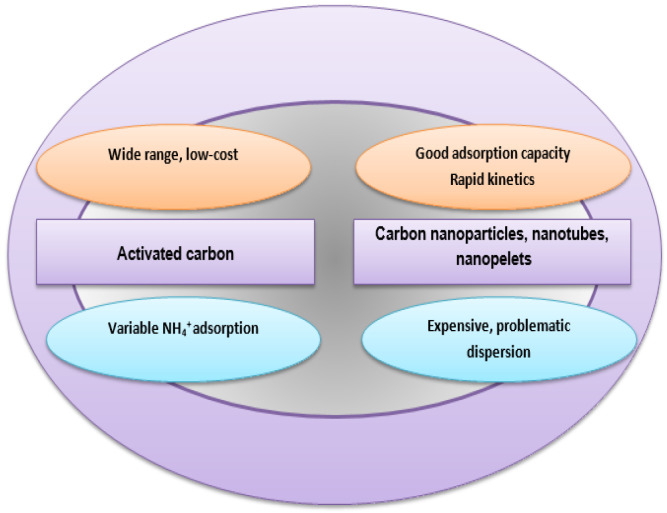
Advantages and disadvantages of different carbon materials as sorbents for ammonium removal from water.

For removing environmental contaminants by using these nanomaterials, whose advantages and disadvantages as sorbents for treating natural waters are presented in Figure 2, the sorption processes are conducted in neutral aqueous media in order to influence as little as possible the ammonium adsorption mechanism, whose elucidation needs a simple chemical composition of the matrices [20]. The role of surface chemistry was confirmed by previous studies for ammonium sorption on carbon nanomaterials [21], but very few articles report data over ammonium adsorption on modified carbon-based nanomaterials, especially by oxidation, these aspects offering a better understanding of the influence of surface modification [22]. 

There are several treatment methods that can be used, such as those presented below in Table 1, but adsorption processes present advantageous features, such high removal efficiency, ease of operation and low energy consumption. Additionally, treatments based on adsorption procedures are the only admitted treatments for natural mineral waters, based on European legislation, as mentioned before.

Membrane purification, photocatalysis and ion exchange methods are indicated treatment methods for ammonium removal from natural waters, but in comparison with the adsorption on activated carbon, all of them need several procedural steps; in the case of membrane filtration, sludge can appear, and photocatalysis has limited application.

The objective of this research was to study the process of ammonium adsorption on carbon-based nanomaterials, by using as sorbent exfoliated graphite nanoplatelets, xGnP, oxidized with HNO_3_ during three hours reflux time. xGnP were chosen due to their square-shaped sheets with dimensions on the micrometer range and thicknesses on the nanometer one, less than 100 nm. These offer facile sites for obtaining oxidized graphite nanoplatelets (ox-xGnP). HNO_3_ was preferred because it involves a single step oxidation process with variable oxidation times. An adsorption study was done controlling the pH of the solution and repeated washing of the sorbents. It is necessary to discriminate between the acidic functional groups obtained through different refluxing times using the same oxidizing reagent (HNO_3_) because it can be presumed that the acidic functional groups control the ion-exchange process of ammonium onto carbon nano sorbents in aqueous media, the process being reversible and of electrostatic nature. This method is simple, offering an economical approach for modifying xGnP.

## 2. Results

Ammonium adsorption on ox-xGnP was studied, considering several factors, such as the characteristics of the nanosheet surface and the composition of the solution (pH value and the concentrations of adsorbate and adsorbent). Ox-xGnP were obtained by oxidation with nitric acid, having observed from the literature that the type and number of acidic sites in oxidized xGnP depend on the oxidation process [29]. The chemical treatment and the refluxing time led to xGnP with different degrees of oxidation, resulting in ox-xGnP with different sorption capacities for ammonium at the same concentration of equilibrium. Lengthening the refluxing time by using 65% HNO_3_ from Fluka, higher adsorption capacities could be observed at the studied temperatures. The oxidized carbon surface showed increased hydrophilicity and increased ion-exchange capability too [30]. The ion-exchange interactions between ammonium and the hydrogen ions from oxygen containing functional groups appeared as the main sorption mechanism, a release of H^+^ ions leading to a decrease of the pH of the solution. Physisorption can also influence ammonium sorption at the xGnP surface based on weak intermolecular physical interactions, such as van der Waals interactions, electrostatic interactions and diffusion, together with hydrophobic interactions too.

### 2.1. Characterization of Oxidized xGnP 

#### 2.1.1. Fourier Transform Infrared Spectra (FTIR) Spectra

**Figure 3:**
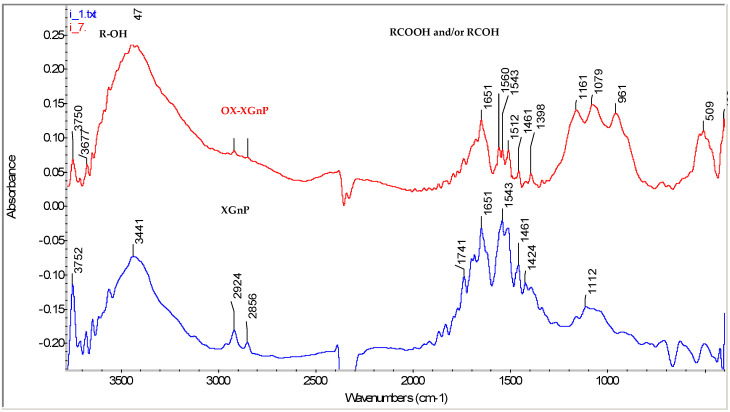
Fourier transformation infrared (FTIR) spectra of as-prepared (blue line) and oxidized xGnP (red line).

To investigate the ox-xGnP structure, Fourier transform infrared spectra (FTIR) of xGnP and ox-xGnP in pressed KBr pellets are presented in Figure 3. 

The absorption spectra showed peaks at 3441 cm^−1^ for xGnP and 3447 cm^−1^ for ox-xGnP, which corresponded to hydroxyl groups HO- from atmospheric natural humidity, these peaks increasing after the oxidation process. After the HNO_3_ treatment, characteristics peaks at 2904 cm^−1^ for xGnP and 2918 cm^−1^ for ox-xGnP are provided by the presence of symmetric and asymmetric -CH_2_ groups. The evidence of -COOH group formation is demonstrated by the new peak appearing at 1161 cm^−1^ for ox-xGnP, corresponding to C-O stretching and at 1398 cm^−1^ coming from the intermediate oxidation products and H-O- bending vibrations. The peak at 1560 cm^−1^ corresponds with C=O groups in ox-xGnP. 

#### 2.1.2. Scanning Electron Microscopy (SEM)

**Figure 4:**
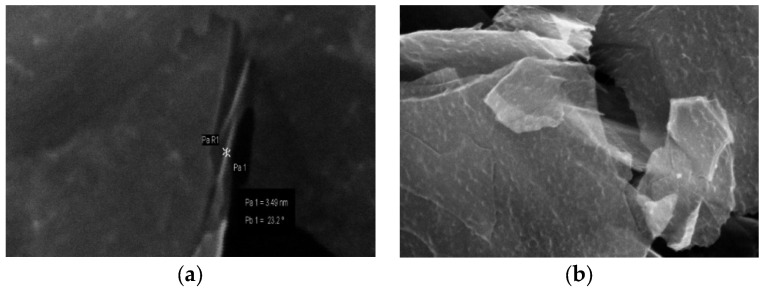
SEM images of xGnP (**a**) and ox-xGnP (**b**).

In the investigations of xGnP microstructure and their visualization, scanning electron microscopy was used (SEM). The surface morphology of ox-xGnP is presented, being observed from Figure 4 that xGnP its thickness is approximately 10 nm, but it could vary, with irregular large shapes of micrometer dimensions. 

After the oxidation of xGnP, oxygen containing groups appeared at the edges of the surfaces (white spots). The fragmentation of the sheets also indicated the oxidation process. The different acidic functionalities at the surface of xGnP, such as carboxyl, carbonyl and phenol, introduced by oxidation, increased their hydrophilic properties, improving the dispersion of ox-xGnP in aqueous media, in comparison with that of xGnP. If the pH of the working solution is higher than the pH of zero charge, the total negative charge of the surface provides electrostatic interactions, favorable for the adsorption of positively charged species.

#### 2.1.3. Thermogravimetric Analysis

**Figure 5:**
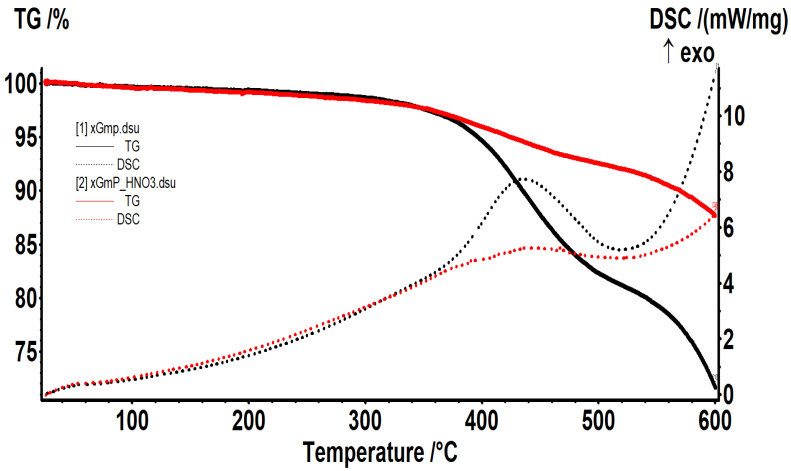
Thermograms of xGnP (black line) and ox-xGnP(red line).

Differences between xGnP and ox-xGnP properties can be observed from Figure 5, where the results obtained by thermogravimetric analysis are presented.

The xGnP sample presented a mass loss of 1.41% up to 300 °C, due to the oxidation of amorphous carbon. Between 300 and 520 °C, the xGnP sample presented a mass loss of 17.48%, together with an exothermic effect with a maximum at 436.2 °C. The onset temperature of 377.3 °C was too high to consider that it was due to the oxidation of amorphous carbon; it was probably due to it being a partial oxidation of exfoliated graphite nanoplatelets. Till 600 °C, the sample lost 9.47% from the initial mass through a new oxidation process that would probably continue by increasing the temperature. After the oxidation treatment by using HNO_3_, the xGnP sample developed several defects in the structure of graphite nanosheets, and by consequence, a 1.77% mass loss till 300 °C. Between 300 and 510 °C, the mass loss was 6.10%, the process being accompanied by an exothermic effect with a maximum at 465.3 °C. The onset temperature of 338.6 °C indicates a structural modification of the graphite nanosheets because of a facilitated oxidation due to lower activation energies. On the other hand, the mass loss was reduced in comparison with the xGnP, this aspect indicating the removal of small fragments after oxidation. Till 600 °C, the sample still lost 4.61% of the initial mass, continuing the oxidation process, without reaching a constant mass. 

#### 2.1.4. Boehm’s Titration

The acidic and basic functional groups were calculated based on Boehm’s titration method. The various acidic functionalities can be identified and quantified by using sodium hydrogen carbonate to neutralize only the carboxyl groups and sodium carbonate to neutralize the carboxyl groups and lactones. Based on this, NaHCO_3_ (pK = 10.25) reacted with carboxylic groups (pK < 5), but not with hydroxyl groups from phenols, or alcohols (pH > 9). Sodium hydroxide neutralized carboxyl groups, lactones and phenols, meaning the acidic sites. Different kinds of acidic functional groups were quantified by using the titration volume of the acid as mmol H_3_O^+^ equivalent/g ox-xGnP. In Table 2, the determined amounts of acidic groups on ox-xGnP obtained by different oxidation methods are shown. 

It can be observed from Table 2 that the concentrations of acidic groups obtained by several oxidation treatments differed based on the conditions of reaction, which was necessary for estimating each of them by several methods, with Boehm’s titration being one such method.

The changes of the specific surface area (S_BET_) and of the pH of point of zero charge (pH_PZC_) were consistent with the literature [34]. The specific surface areas were calculated based on adsorption/desorption data, using Brunauer, Emmet, Teller (BET) equations. The specific surface area was measured by nitrogen adsorption–desorption using the BET method at a relative pressure (P/P0) range of 0.0001–0.99 [35]. Based on the BET equation, the specific surface was calculated, resulting in a specific surface area that was higher for the ox-xGnP obtained by oxidation with 65% HNO_3_, for three hours reflux time. 

**Figure 6:**
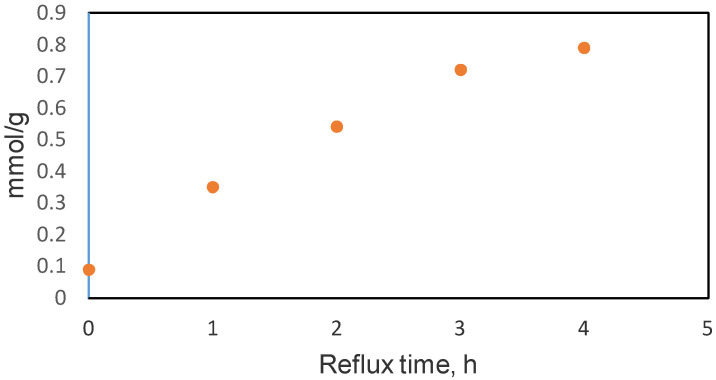
Correlation between the calculated amounts of carboxylic groups vs. reflux time (total reflux time 4 h).

The concentration of the carboxylic sites increased during the three hours refluxing time, probably because these acidic functional groups were evolved from the ketone ones. Based on Boehm’s titration, which quantifies the concentration of carboxylic groups depending on the different refluxing times in the oxidation process using HNO_3_, it seems that their number could be improved till a certain value of the reflux time of three hours, as can be observed in Figure 6. Longer refluxing times led to worsened oxidation, possibly based on the location of acidic groups at the edges of the nanosheet layers and their transformation into CO_2_ during longer reflux treatment.

It was observed that the oxidation of xGnP by using 65% HNO_3_ and refluxing the mixture for three hours increased the number of acidic functional groups, with the carboxyl groups being higher in comparison with the HNO_3_ treated xGnP that was refluxed for one hour. 

**Figure 7:**
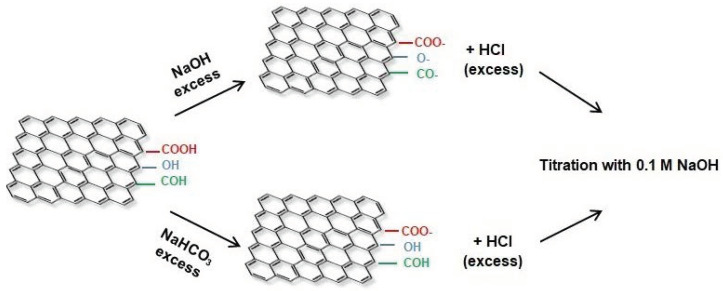
Scheme of the analytical method developed to titrate selectively the total acidic sites and the carboxylic groups created at MWCNT surface.

For determining the total acidic sites, the steps are presented in Figure 7. Known volumes of known concentration of NaOH were added, reacting with the acid groups at the xGnP surface. Further on, known volumes of 0.1 M HCl were added, the excess of HCl being titrated with NaOH of exact concentration. Then each oxygen containing group was determined by following the same procedure but choosing the appropriate solution in excess. For the determination of the carboxylic groups, known volumes of known concentration of NaHCO_3_ were added instead of NaOH, which reacted with the -COOH groups due to the low values of pK_a_ in comparison with the other resulting acidic surface groups. The remaining bicarbonate ions, after the reaction was completed, reacted with an equivalent volume of HCl initially added to the volume of NaHCO_3_, and the excess of HCl was titrated with NaOH as titration reagent, being equivalent to the quantity of carboxylic groups at the ox-xGnP surface. 

## 3. Discussion

### 3.1. Adsorption Studies

#### 3.1.1. Kinetic Results

The kinetic models used to study the adsorption kinetics onto ox-xGnP were pseudo-first-order, pseudo-second-order and intra-particle diffusion models. In all investigations of the kinetics of the sorption mechanism, the experimental data were evaluated especially based on pseudo-first- and pseudo-second-order kinetic models [36].

For the first model, the Lagergreen equation is:

(1) $$\ln(q_{e}-q_{t})=\ln q_{e}-k_{1}t$$

where: *q_t_* is the amount of ammonium adsorbed on ox-xGnP at time *t*, (mg g^−1^), *q_e_* is the amount of ammonium adsorbed on ox-xGnP at equilibrium (mg g^−1^), *k*_1_ is the kinetic constant of the pseudo-first-order kinetic model (min^−1^) obtained from linear correlation ln(*q_e_ − q_t_)* vs. *t* (contact time, min).

The experimental data were analyzed by the pseudo-second-order kinetic model, as follows:

(2) $$\frac{t}{q_{t}}=\frac{1}{k_{2}q_{e}^{2}}+\frac{t}{q_{e}}$$

where: *q_t_* is the amount of ammonium adsorbed on ox-xGnP at time *t*, (mg g^−1^), *q_e_* is the amount of ammonium adsorbed at equilibrium (mg g^−1^), *k*_2_ is the rate constant of the pseudo-second-order kinetic model (g mg^−1^ min^−1^) obtained from the correlation t/*q_t_* vs. *t*, *t*—contact time (min.). 

The intra-particle diffusion model has the following equation:

(3) $$q_{t}=k_{\mathrm{id}}t^{1/2}+C$$

where: *q_t_* is the amount of ammonium adsorbed on ox-xGnP at time *t*, (mg g^−1^), *k_id_* is the intra-particle diffusion constant (mg g^−1^ min^−1/2^) obtained from the correlation *q_t_* vs. *t*^1/2^, *t* is the contact time (min^1/2^), *C* is the concentration of the sorbate at any given time *t* (mg g^−1^).

The evaluation of the kinetic models was done based on the value of *R*^2^, better values being obtained for pseudo-second-order kinetic model. It was also noticed that the experimental value of the sorption capacity was closer to that calculated by the pseudo-second-order kinetic model, being concluded that the sorption process followed this model better, as it is presented in Table 3. Both chemical and physical interactions play an important role at the surface, while the rate determining step seems to be the diffusion process: boundary layer diffusion, liquid film diffusion and internal diffusion, based on the small dimensions of ammonium ions.

Alshameri [39], considered that a well-fitted pseudo-second-order model involves ammonium movement to the solid interface, its diffusion from the liquid to the solid interface and physical and chemical interactions at the surface. Ion-exchange is important, being possible to represent the rate-determining step, but ion-exchange processes are based on electrostatic interactions, while acidic surface functional groups include covalent and ligand bonding too.

#### 3.1.2. Adsorption Isotherm Study

The sorption isotherms describe the way in which ammonium ions interact with the surface of ox-xGnP, offering information about the type of interactions and their intensity. To describe the effect of these interactions over the adsorption process, Freundlich and Langmuir models were applied. The Langmuir model regards the adsorption of a monolayer on the homogeneous surface of the adsorbent, based on the linear and non-linear forms below:

(4) $$q_{e}=\frac{q_{m}K_{L}C_{e}}{1+K_{L}C_{e}},$$

(5) $$\frac{C_{e}}{q_{e}}=\frac{1}{q_{m}}C_{e}+\frac{1}{q_{m}K_{L}},$$

An important parameter is the value of R_L_: R_L_ = 1/(1 + *K_L_C*_o_). The values of the Langmuir isotherm equilibrium parameter R_L_ show unfavorable adsorption (R_L_ > 1), favorable adsorption (R_L_ < 1), linear adsorption (R_L_ = 1) and irreversible adsorption (R_L_ < 0) [40]. 

The Freundlich model is based on the multi-layer adsorption on the surface of the adsorbent, being an empirical model, whose calculated constants that characterize it are *K_F_* and *n*.

(6) $$\log q_{e}=\frac{1}{n}\log C_{e}+\log K_{F}$$

where *q_e_* is the amount of absorbate in the absorbent at equilibrium (mg/g), *q_m_* is the maximum monolayer coverage capacity (mg/g), *C_e_* is the equilibrium concentration (mg/L), *K_L_* is the Langmuir isotherm constant (dm^3^/mg), *n* is the adsorption intensity, T is the temperature (K), *K_F_* is the Freundlich isotherm constant (mg/g), R is the universal gas constant (8.314 J/mol K).

Freundlich constant *n* represents in our case the intensity of cation exchange, values of *n* > 1 meaning higher intensity and facilitated chemisorption [31]. 

**Figure 8:**
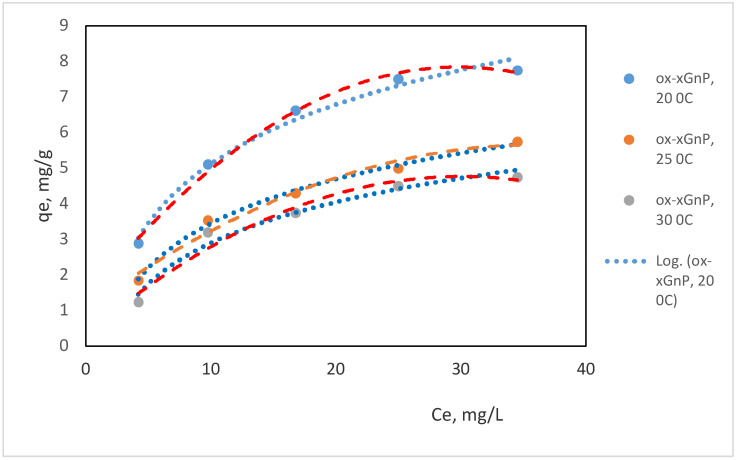
Isotherm models fitting for ammonium adsorption by oxidized xGnP treated with 65 % HNO_3_, three hours reflux time; … Freundlich model, ---Langmuir model and •experimental data at the three temperatures 20, 25 and 30 °C.

All isotherm experiments were conducted at equilibrium time of three hours, solution volume of 50 mL, varying initial concentration ranges from 20 to 40 mg/L NH_4_^+^, at a dosage of 0.1 mg for the carbon nanomaterials in all the sorption experiments, at three temperatures: 20, 25 and 30 °C, as presented in Figure 8.

Ion-exchange processes of ammonium ions with the H^+^ ones, ionized from the acidic surface functional groups are present together with the adsorption on hydrophobic sites, driven by intermolecular forces, but ion exchange is also influenced by diffusion processes. It can be noticed that *n* values increase by temperature; consequently, their reverse values 1/*n* decrease. This aspect proves a non-facilitated adsorption process by increasing the temperature, based on the values presented in Table 4.

**Figure 9:**
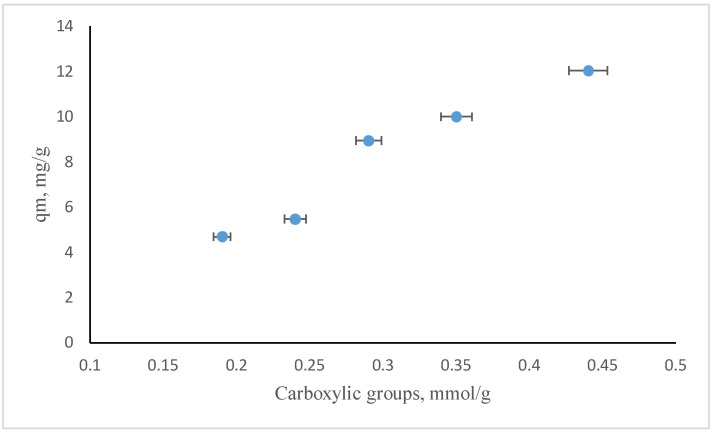
Correlation between the concentration of carboxylic groups at the surface of ox-xGnP and the adsorption capacity of the carbon sorbents.

From the comparison of the isotherm parameters presented in Table 5, it can be noticed that the Langmuir model fits better on carbon-based nanomaterials used as sorbents for ammonium, based on the values of the correlation coefficients. The sorption process takes places at the surface of the nanomaterial based on hydrophobic interactions, such as covalent and ligand bonding at the surface of the sorbent and electrostatic interactions/ion-exchange at the acidic functional groups introduced by oxidation. Concerning the values of *q_m_*, by increasing the number of acidic surface functional groups, the values of maximum adsorption capacity increase, as shown in Figure 9, thus proving that the acidic surface functional groups control the sorption process of ammonium onto carbon-based nanomaterials in aqueous solutions.

There were studies that showed that Langmuir and Freundlich models fit better for ammonium adsorption on carbon-based nanomaterials [46]. Our study showed the same: The R_L_ values determined for xGnP oxidized with 65% HNO_3_, refluxing the mixture for three hours, were <1, meaning favorable ammonium adsorption at the given experimental conditions. For the studied nanomaterials, n values were larger than 1, ion-exchange processes being the most important mechanism of adsorption of ammonium. 

#### 3.1.3. Adsorption Mechanism

**Figure 10:**
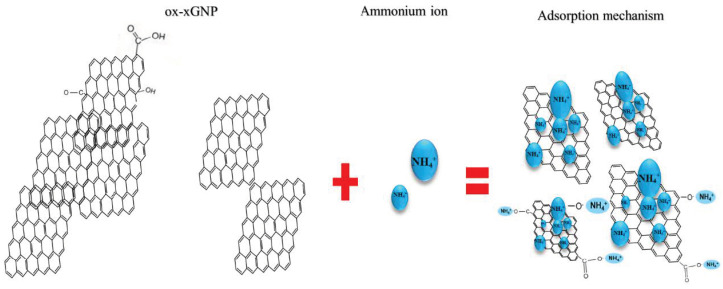
The adsorption mechanism of ammonium ions on ox-xGnP.

The surface of xGnP is supposed to be flat, with the large layers of carbon containing delocalized π electrons, explaining several retention mechanisms such as: electron transfer, ion-pairing and hydrophobic interactions. The adsorption capacity of activated carbon has already been proved as being modified by oxidation [47]; the functional groups that were introduced increased the adsorption capacity, as can be observed in Figure 10. It can be supposed that the number of adsorbed ammonium ions depends on the nature and quantity of the surface acid–base and ion radical functionalities as well as on the pH of the aqueous solution, together with the hydrophobic interactions at the surface of carbon nanomaterial, ion-exchange, physisorption and chemisorption processes being involved.

xGnP might be used as adsorbents, not only for ammonium, but also for several other positively charged species, which are also present at different concentrations in natural mineral waters. Dominant interactions that can appear are physical adsorption of the ions (dipole–dipole interactions) and hydroxy complexes, ion-exchange processes based on electrostatic interactions and chemisorption processes involving the participation of acidic surface groups, which can be involved in covalent and ligand bonding, too, in connection with the type and concentration of the different oxidizing reagents [48].

## 4. Materials and Methods

### 4.1. Chemical Reagents and Instrumentation

Graphite nanoplatelets xGnP were purchased from XG Sciences Inc. (USA). The surface of xGnP was chemically activated with NaOH 0.1 M at 100 °C [49]. The oxidized nanoplatelets were obtained by using 65% HNO_3_ from Fluka. Reagent water: ultrapure water (UPW) from Millipore Direct Q3 with resistivity >18.2 MΩ cm (25 °C). Ammonium chloride salt (NH_4_Cl), concentrated HNO_3_, 0.1 M picolinic acid stock standard anion solution and 1.000 mg L^−1^ stock solution were purchased as certified solutions, or were prepared from ACS reagent grade from Merck. The 65% HNO_3_ was bought from Fluka.

Sonic Vibracell ultrasonic processor, Sonics&Materials, Newtown CT, 06470 USA.

Ion chromatograph: 850 Professional IC (ion chromatography) AnCat-MCS using a Metrohm intelligent Partial Loop (MiPT) technique with conductivity detector; cation chromatographic column-Metrosep C4-150/4.0; cation guard column-Metrosep RP 2 Guard/3.5; Detector: 850 Professional conductivity detectors (0–15,000 µS/cm); professional drift <0.2 nS cm/h; Professional sample processor; and Metrohm patented Dosino technology, Metrohm, Herisau, Switzerland.

FTIR Spectrum GX, Perkin Elmer (Waltham, MA, USA) spectrometer.

(SEM) FEI QUANTA 200 (FEI Company, Hillsboro, OR, USA) scanning electron microscope.

Quantachrome NOVA 2200e (Boynton Beach, FL, USA) instrument.

### 4.2. Oxidation of xGnP

For oxidation treatment, the graphite nanoplatelets (XG Sciences) were refluxed with HNO_3_ 65% Fluka, for three hours. As-grown xGnP was added and mixed for one hour at 80 °C, then refluxed for three hours at 140 °C. These were washed after with ultrapure water to remove the free oxidants, dried at 80 °C and stored at 4 °C between experiments [50]. After the oxidation treatment with 65% HNO_3_, the acidic functionalities located at the edges modified the Van der Waals interactions between the sheets of graphite oxide, facilitating the determination of exfoliation.

### 4.3. Batch Sorption Experiments

Batch adsorption experiments were conducted using 100 mL glass vials; in each 0.1 mg of sorbent was accurately weighed, and 50 mL of ammonium salt solution with a certain initial concentration C_o_ less than 40 mg/L was added. The concentration of ammonium was chosen taking into account its concentrations in ground and underground waters. The glass vials were sealed and mounted in a 750 Watt thermostat ultrasonic processor; the adsorption process was tested at several temperatures between 20 and 35 °C. The glass vials did not show any adsorption of ammonium after testing. In all experiments, the initial pH was checked, but it was not adjusted before the experiments because the values were in the range of 5.5–8.0, and they did not influence the adsorption capacity of the ox-xGnP (*q_e_*, mg/g), calculated as: *q_e_* = (*C_o_* − *C_e_*) × *V*/*m*, where *C_o_* and *C_e_* (mg/L) are the initial and equilibrium concentrations, respectively, *V* is the initial solution volume (L) and *m* is the weight of adsorbent (g). The adsorption equilibrium *q_e_* was considered at the steady state, reached after a certain checked time, and then calculated. The procedure is described in more detail in [41]. The graphite nanoplatelets were better dispersed by activation before the sorption process, xGnP with a NaOH solution, followed by several washing steps [18].

### 4.4. Analytical Methods

Analytical standardized IC method SREN ISO 14911 was used for the ammonium determinations in all the aqueous solutions. The procedure is described in [51].

The BET surface of xGnP and ox-xGnP was characterized from the nitrogen adsorption at the working temperature of 77 K, at which the adsorption–desorption isotherms were registered, the samples being degassed in vacuum at 60 °C for 4 h.

Carbon nanomaterials characterization was done by Fourier transformed infrared spectroscopy (FTIR) analysis and scanning electron microscopy (SEM). Fourier transform infrared (FT-IR) spectra of ox-xGnP were registered on KBr pellets, with a resolution of 4 cm^−1^. 

The type and concentration of acidic surface functional groups obtained by oxidation were determined by using Boehm’s titration as follows: 20 mg ox-xGnP was stirred in 10 mL of 0.05 M NaOH, Na_2_CO_3_ and NaHCO_3_ aqueous solution for 48 h, under inert atmosphere. After filtration on 0.20 µm pore size Millipore filters, volumes of 10 mL of each mixture were titrated with 0.05 M HCl, as follows: three aliquots of each NaOH, Na_2_CO_3_ and NaHCO_3_ solution were titrated in parallel with a blank that did not contain xGnP. The NaOH solution neutralized carboxyl, lactone and phenol groups; the NaHCO_3_ solution only neutralized carboxyl groups and Na_2_CO_3_ reacted with carboxyl and lactone groups. The quantitation of each group was possible based on the difference between the calculated amounts of each functional group. 

The pH of the point of zero charge (pH_PZC_) was determined based on the pH drift method [52]. 

## 5. Conclusions

Removal of ammonium ions from natural mineral waters was done by using oxidized exfoliated carbon nanoplatelets (ox-xGnP), oxidized by refluxing for three hours with 65% HNO_3_. Ox-xGnP proved to be good adsorbents for ammonium ions, based on the type and concentration of oxygen-containing groups at the carbon surface. The maximum sorption capacity was of 12.04 mg g^−1^, based on Langmuir model for sorption isotherm. Langmuir isotherm dimensionless equilibrium parameter R_L_ values were between 0.42 and 0.49, meaning favorable ammonium adsorption under the given experimental conditions. Freundlich constant n showed favorable chemisorption, having values higher than 1 in the sorption experiments. 

Based on the obtained results, the sorption mechanisms involved in removing ammonium by oxidized carbon nanomaterials combine ion-exchange, chemisorption and hydrophobic interactions, conferring to these sorbents a multifunctional character that can possibly be exploited for the simultaneous removal of several species from natural waters, such as radionuclides, heavy metals and organics, also present in these aqueous matrices.

## Figures and Tables

**Table 1:** Admitted approaches for ammonium removal from natural waters.

Approach	Conditions	Strengths	Weaknesses	Removal Efficiency, %	Ref.
Adsorption	Different temperatures and pH values	Simple equipment	Based on the adsorbent	43–100	[23]
Membrane Purification	pH: 8–12 temp. >15 °C	Applied in complex matrices	Energy and time consuming	50–90	[24]
Photocatalysis	In liquid phase	Sustainable method	Variable efficiency	35–80	[25]
Ion exchange		Waste materials	Variable efficiency	50–100	[26]
Combined methods	Different reaction mechanisms are involved	Limitations of each mechanism are balanced by advantages of other components	Complex studies are developed by using new multi-functional nano-sorbents	Variable	[27,28]

**Table 2:** Specific surface area, pH of the point of zero charge and the number of functional groups on the surface of oxidized xGnP (ox-xGnP) determined by Boehm titration.

Sample	Specific Surface ^1^ Area SBET, m^2^ g^−1^	pH of the Point of Zero Charge, pH_PZC_^2^	Carboxyl ^3^ (mmol g^−1^)	Lactone ^3^ (mmol g^−1^)	Phenol ^3^ (mmolg^−1^)	Ref.
As-grown xGnP	110	8.5	-	-	-	[31]
HNO_3_ oxidized xGnP, reflux time one hour	174	2.7	0.35	1.40	1.72	[32]
KMnO_4_ oxidized xGnP	153	4.3	0.87	1.26	1.09	[33]
NaOH treated xGnP	135	8.9	0.05	0.03	0.02	[32]
65% HNO_3,_ reflux time 3 h, oxidized xGnP	208	5.8	0.44	1.25	0.31	This paper

^1^ SBET, m^2^ g^−1^, specific surface area. ^2^ pH_PZC_, pH of the point of zero charge. ^3^ Carboxyl, lactone, phenol groups present the number of functional groups on the surface of oxidized xGnP, as determined by Boehm’s titration.

**Table 3:** Pseudo-first-order and pseudo-second-order kinetic models for the removal of NH_4_^+^.

Sorbent, (25 °C)	*q_e_*_exp._ (mg g^−1^)	The First-Order Kinetic Model			The Second-Order Kinetic Model			Intra-Particle Diffusion Model			Ref.
		*q_e_*_cal._ (mg g^−1^)	k_1_ (min^−1^)	*R* ^2^	*q_e_*_cal._ (mg g^−1^)	k_2_ (g mg^−1^ min^−1^)	*R* ^2^	k_id_ (mg g^−1^ min^−1/2^)	C (mg g^−1^)	*R* ^2^	
Lignite	3.43	1.31	0.533	0.7080	0.33	5.949	0.999	1.64	1.17	0.9780	[37]
GO	6.6	3.53	0.071	0.9574	6.95	0.037	0.9967				[38]
Ox-xGnP	6.15	1.76	0.035	0.9380	1.47	0.021	0.9900	0.2604	3.01	0.9750	[32]
xGnP	5.65	4.14	0.042	0.9826	2.79	0.001	0.9831				[31]
Ox-xGnP This paper	6.76	4.43	0.056	0.9238	5.26	0.046	0.9964	0.922	0.58	0.9976	This work

**Table 4:** The values of isotherm parameters for ammonium adsorption on ox-xGnP at different temperatures.

T (K)	Freundlich				Langmuir			
	Parameter			Performance	Parameter			Performance
	*K_F_*	*n*	1/*n*	*R* ^2^	*K_L_* (L mg^−1^)	*q_m_* (mg g^−1^)	R_L_	*R* ^2^
293	1.91	1.45	0.41	0.9851	0.0736	12.04	0.49	0.9166
298	0.79	1.51	0.54	0.9992	0.0346	10.51	0.45	0.9922
303	0.58	1.50	0.58	0.9652	0.0373	8.17	0.42	0.9768

**Table 5:** A comparison of isotherm parameters of Freundlich and Langmuir models for NH_4_^+^ adsorption from the literature.

Sorbent	T °C	Freundlich			Langmuir			Ref.
		1/*n*	*K_F_* (mg g^−1^)	*R* ^2^	*q_m_* (mg g^−1^)	*K_L_* (L mg^−1^)	*R* ^2^	
NaOH Lignite	20	0.43	0.17	0.9570	0.67	0.392	0.997	[32]
NaOH AC	20	2.05	1.81	0.9650	17.03	0.039	0.9800	[5]
Coconut shell AC	25	0.75	0.04	0.9800	5.47	0.003	0.9800	[41]
AC	20	1.34	0.03	0.9800	5.47	0.003	0.9800	[42]
ox xGnP	25	0.3891	1.44	0.9649	9.41	0.051	0.9903	[12]
xGnP	25	1.6037	1.06	0.9897	1.66	0.025	0.9987	[43]
HNO_3_ ox xGnP	25	0.64	0.12	0.9940	10.00	0.079	0.9920	[44]
NaOH xGnP		0.57	0.58	0.9764	8.17	0.037	0.9743	[33,45]
65% HNO_3,_ reflux time 3 h ox-xGnP	20	0.41	1.91	0.9851	12.04	0.076	0.9768	This paper

## Data Availability

The data presented in this study are available on request from corresponding author.

## Sample Availability

Not available.

## References

[1] Olariu A., Palcu M. (2019). The origin of ammonium in carbonated mineral waters and its underground transport to one production well in Middle Ciuc Depression from Eastern Carpathians. Int. Symp. Environ. Ind..

[2] Katz M.J., Howarth A.J., Moghadam P.Z., DeCoste J.B., Snurr R.Q., Hupp J.T., Farha O.K. (2016). High volumetric uptake of ammonia using Cu-MOF-74/Cu-CPO-27. Dalton Trans..

[3] European Parliament, Council of the European Union (2009). Directive 2009/54/EC of the European Parliament and of the Council of 18 June 2009 on the exploitation and marketing of natural mineral waters. Off. J. Eur. Union.

[4] Singh N.B., Nagpal G., Agraval S., Rachna (2018). Water purification by using Adsorbents: A Review. Environ. Technol. Innov..

[5] Vu M.T., Chao H.P., Van Trinh T., Let T.T., Lin C.C., Tran H.N. (2018). Removal of ammonium from groundwater using NaOH treated activated carbon derived from corncob wastes: Batch and column experiments. J. Clean. Prod..

[6] Calin M.R., Ion A.C., Radulescu I. (2015). Evaluation of quality parameters and of natural radionuclides concentrations in natural mineral water in Romania. J. Radioanal. Nucl. Chem..

[7] Zou Y., Wang X., Ai Y., Liu Y., Ji Y., Wang H., Hayat T., Alsaedi A., HU W., Wang X. (2016). β-Cyclodextrin modified graphitic carbon nitride for the removal of pollutants from aqueous solution: Experimental and theoretical calculation study. J. Mater. Chem. A.

[8] Zou Y., Wang X., Ai Y., Liu Y., Li J., Ji Y., Wang X. (2016). Coagulation Behavior of Graphene Oxide on Nanocrystallined Mg/Al Layered Double Hydroxides: Batch Experimental and Theoretical Calculation Study. Environ. Sci. Technol..

[9] Zou Y., Wang X., Khan A., Wang P., Liu Y., Alsaedi A., Hayat T., Wang X. (2016). Environmental Remediation and Application of Nanoscale Zero-Valent Iron and Its Composites for the Removal of Heavy Metal Ions: A Review. Environ. Sci. Technol..

[10] Yan T., Li T.X., Wang R.Z., Jia R. (2015). Experimental investigation on the ammonia adsorption and heat transfer characteristics of the packed multi-walled carbon nanotubes. Appl. Therm. Eng..

[11] Yang H., Zuttel H., Kim S., Ko Y., Kim W. (2017). Effect of Boron Doping On Graphene Oxide for Ammonia Adsorption. ChemNanoMat.

[12] Ion I., Culetu A., Gherase D., Sarbu F., Ion A.C., Ion A.C., Dascalu D., Carja C. (2014). Environmental applications of carbon based nanomaterials II. Micro and Nanoengineering.

[13] Chim Y.J., Shin T.S., Choi H.D., Kwon J.J., Chung Y., Yoon H.G. (2005). Electrical conductivity of chemically modified multiwalled carbon nanotube/epoxy composites. Carbon.

[14] Kim Y., Mitani T. (2006). Competitive effect of carbon nanotubes oxidation on aqueous EDLC performance: Balancing hydrophilicity and conductivity. J. Power Sources.

[15] Allen B.L., Kichambare P.D., Star A. (2007). Carbon Nanotube Field-Effect-Transistor-Based Biosensors. Adv. Mater..

[16] Murphy H., Papakonstantinou P., Okpalugo T.I.T. (2006). Raman study of multiwalled carbon nanotubes functionalized with oxygen groups. J. Vac. Sci. Technol. B.

[17] Mawhinney D.B., Naumenko V., Kuznetsova A., Yates J.T., Liu J., Smiley R.E. (2000). Surface defect site density on single walled carbon nanotubes by titration. Chem. Phys. Lett..

[18] Li Y., Pu Z., Sun Q., Pan N. (2021). A review on novel activation strategy on carbonaceous materials with special morphology/texture for electrochemical storage. J. Energy Chem..

[19] Gonzales-Guerrero A.B., Mendoza E., Pellicer E., Alsina F., Fernandez-Sanchez C., Lechuga L.M. (2008). Discriminating the carboxylic groups from the total acidic sites in oxidized multi-wall carbon nanotubes by means of acid-base titration. Chem. Phys. Lett..

[20] Khalil A., Seergeevich S.N., Borisova V. (2018). Removal of ammonium from fish farms by biochar obtained from rice straw: Isotherm and kinetic studies for ammonium adsorption. Adsorpt. Sci. Technol..

[21] Tang Y., Alam M.S., Konhauser K.O., Alessi D.S., Xu S., Tian W., Liu Y. (2019). Influence of the pyrolysis temperature on production digested sludge biochar and its application for ammonium removal from municipal wastewater. J. Clean. Prod..

[22] Fan R., Chen C.L., Li Y.J., Tzeng J.H., Huang C.P., Dong C. (2019). Adsorption characteristics of ammonium ion onto hydrous biochars in dilute aqueous solutions. Bioresour. Technol..

[23] Han B., Butterly C., Zhang W., He J., Chen D. (2021). Adsorbent materials for ammonium and ammonia removal: A review. J. Clean. Prod..

[24] Tajdemir A., Cengiz I., Yildiz E., Bayhan Y.K. (2020). Investigation of ammonia stripping with a hydrodynamic cavitation reactor. Ultrason. Sonochem..

[25] Ren H.T., Liang Y., Han X., Liu Y., Wu S.H., Bai H., Jia S.Y. (2020). Photocatalytic oxidation of aqueous ammonia by Ag_2_O/TiO_2_ (P25): New insights into selectivity and contributions of different oxidative species. Appl. Surf. Sci..

[26] Guadayol M., Cortina M., Guadayol J.M., Caixhah J. (2016). Determination of dimethyl selenide and dimethyl sulphide compounds causing off-flavours in bottled mineral waters. Water Res..

[27] Luo Z., He Y., Zhi D., Luo L., Sun Y., Khan E., Wang L., Peng Y., Zhou Y., Tsang D.C.W. (2019). Current progress in treatment techniques of triclosan from wastewater: A review. Sci. Total Environ..

[28] Zhang P., Zeng X.Z., Wen X.H., Yang C.K., Ouyang S.D., Li P., Gu Z., Wu D.S., Frost R.L. (2019). Insights into efficient removal and mechanisms for ammonium from aqueous solution on trcicalcium aluminate. Chem. Eng. J..

[29] Boehm H.P. (1994). Some aspects of the surface chemistry of carbon blacks and other carbons. Carbon.

[30] Gao F., Xue Y., Deng P., Cheng X., Yang K. (2015). Removal of aqueous ammonium by biochars derived from agricultural residuals at different pyrolysis temperatures. Chem. Spec. Bioavailab..

[31] Foo K.Y., Hammed B.H. (2018). Insights into the modelling of adsorption isotherms systems. Chem. Eng. J..

[32] Nazari M.A., Mohaddes F., Pramanik B.K., Othman M., Muster T., Bulyan M.A. (2018). Application of victorian brown coal for removal of ammonium and organics from wastewater. Environ. Technol..

[33] Ion A.C., Ion I., Culetu A. (2011). Lead adsorption onto exfoliated graphitic nanoplatelets in aqueous solutions. Mater. Sci. Eng. B.

[34] Gusain R., Kumar N., Ray S.S. (2020). Recent advances in carbon-nanomaterial-based adsorbents for water purification. Coord. Chem. Rev..

[35] Quach N.K.N., Yang W.D., Chung Z.J., Tran H.L. (2017). The influence of the activation temperature on the structural properties of the activated carbon xerogels and their electrochemical performance. Ann. Mater. Sci. Eng..

[36] Kizito S., Wu S., Kirui W.K., Lei M., Lu Q., Bah H., Dong R. (2015). Evaluation of slow pyrolyzed wood and rice husks biochar for adsorption of ammonium nitrogen from piggery manure and anaerobic digestate slurry. Sci. Total Environ..

[37] Tu Y.N., Feng P., Ren Y.G., Cao Z.H., Wang R., Xu Z.Q. (2019). Adsorption of ammonia nitrogen on lignite and its influence on coal water slurry preparation. Fuel.

[38] Wu C., Zhang X., Li C., Cheng C., Zheng Y. (2018). Adsorption of ammonium by graphene oxide based composites prepared by UV irradiation and using a slow-release fertilizer. J. Polym. Environ..

[39] Alshameri A., He H.P., Zhu J.X., Xi Y.F., Zhu R.L., Ma L.Y., Tan Q. (2018). Adsorption of ammonium by different natural clay minerals: Characterization, kinetics and adsorption isotherms. Appl. Clay Sci..

[40] Reguyal F., Sarmah A.K., Gao W. (2017). Synthesis of magnetic biochar from pine sawdust via oxidative hydrolysis of FeCl_2_ for the removal sulfamethoxazole from aqueous solution. J. Hazard. Mater..

[41] Rambabu N., Rao B.V.S.K., Surisetty V.R., Das U., Dalai A.K. (2015). Production, characterization and evaluation of activated carbon from de-oiled canola meal for environmental applications. Ind. Crop. Prod..

[42] Sumaraj X., Sarmah A.K., Padhye L.P. (2020). Acidic surface functional groups control chemisorption of ammonium onto carbon materials in aqueous media. Sci. Total Environ..

[43] Bogdan D., Rizea G.A., Ion I., Ion A.C. (2016). Ammonium adsorption on exfoliated graphite nanoplatelets. Rev. Chim..

[44] Bogdan D., Muklive A.J.S., Ion I., Ion A.C. (2017). Ammonium adsorption on oxidized exfoliated graphite nanoplatelets. Environ. Eng. Manag. J..

[45] Mathurasa S., Damrongsiri S. (2018). Low cost and easy rice husk modification to efficiently enhance ammonium and nitrate adsorption. Int. J. Recycl. Org. Waste Agric..

[46] Gai X., Wang H., Liu J., Zhai L., Liu S., Ren T., Liu H. (2014). Effects of feedstock and pyrolysis temperature on biochar adsorption of ammonium and nitrate. PLoS ONE.

[47] Zhou Z., Zhang Z., Peng H., Qin Y., Li G., Chen K. (2014). Nitrogen and oxygen-containing activated carbon nanotubes with improved capacitive properties. RSC Adv..

[48] Valcarcel M., Cardenas S., Simonet B.M., Moliner-Martinez Y., Lucena R. (2008). Carbon nanostructures as sorbent materials in analytical processes. Trends Anal. Chem..

[49] Gerasimova A., Smolzky G., Melezhyc A., Galunin E., Memetov N., Tkakhev A. Synthesis, Study and Applications of Graphene Materials. Proceedings of the 4th World Congress on Recent Advances in Nanotechnology (RAN’19), ICNNFC 103.

[50] Assis L.K., Damasceno B.S., Carvalho M.N., Oliveira E.H.C., Ghislandi M.G. (2020). Adsorption capacity comparison between graphene oxide and graphene nanoplatelets for the removal of coloured textile dyes from wastewater. Environ. Technol..

[51] Ion I., Bogdan D., Ion A.C. (2014). Improvement in the determination of traces of nitrate and nitrite in natural mineral waters by ion chromatography. UPB Sci. Bull. Ser. B Chem. Mater. Sci..

[52] Chen J., Lu H., Chen Y., Tao Z., Shao M. (2017). Stable aqueous dispersion of polymer functionalized graphene sheets from electrochemical exfoliation for anticorrosion application. Colloid Polym. Sci..

